# Spinal Cord Lesion by Minor Trauma as an Early Sign of Multiple System Atrophy

**DOI:** 10.3389/fneur.2016.00033

**Published:** 2016-03-14

**Authors:** Marisa Brum, Sofia Reimão, Djalma Sousa, Rui de Carvalho, Joaquim J. Ferreira

**Affiliations:** ^1^Campus Neurológico Sénior (CNS), Torres Vedras, Portugal; ^2^Department of Neurology, Hospital São Bernardo, Centro Hospitalar de Setúbal, Setúbal, Portugal; ^3^Clinical Pharmacology Unit, Instituto de Medicina Molecular, Lisbon, Portugal; ^4^Department of Neuroimaging, Centro Hospitalar Lisboa Norte, Hospital de Santa Maria, Lisbon, Portugal; ^5^Department of Neurosurgery, CUF Clinic, Torres Vedras, Portugal; ^6^Laboratory of Clinical Pharmacology and Therapeutics, Faculty of Medicine, University of Lisbon, Lisbon, Portugal

**Keywords:** spinal cord lesion, multiple system atrophy, trauma, early sign, atypical parkinsonism

## Abstract

Multiple system atrophy (MSA) is characterized clinically by parkinsonism, cerebellar, autonomic, and corticospinal features of variable severity. When the presentation is only parkinsonism, the disease might be difficult to differentiate from Parkinson’s disease (PD). We present a case of an 80-year-old man with previous diagnosis of PD. One year after the diagnosis, he had a whiplash cervical trauma due to a tricycle accident caused by a hole in the road. This low-energy trauma caused an unstable C4–C5 cervical fracture with spinal cord injury, which required surgical decompression and stabilization. Neurological examination showed marked postural instability, no rest and postural tremor, finger tapping slowed on the right, spastic tetraparesis (ASIA D) – predominantly on the left side, brisk deep tendon reflexes in the upper and lower extremities, and bilateral extensor plantar response. He also presented with vertical gaze restriction, mild hypometria in horizontal saccades, moderate dysphagia, and dysphonia. As atypical parkinsonism was suspected, he underwent an MRI that revealed conjunction of findings suggestive of parkinsonian-type MSA. In our case, we hypothesize that the loss of postural reflexes, as an early manifestation of MSA, did not allow the patient to have an effective reaction response to a low-energy trauma, resulting in a more severe injury. With this case report, we speculate that the severe spinal lesions caused by minor accidents can be an early sign of postural instability, which may lead to clinical suspicion of neurodegenerative disorder manifested by postural reflexes impairment.

## Introduction

We describe the case of an 80-year-old man with previous diagnosis of Parkinson’s disease (PD). The first symptom of the disease was rest tremor in the right upper limb. Initial treatment with levodopa/carbidopa (300 mg/day) resulted in poor clinical response. One year following the diagnosis, he suffered a whiplash cervical trauma due to a tricycle accident caused by a hole in the road. This low-energy trauma caused an unstable C4–C5 cervical fracture with spinal cord injury, which required surgical decompression and stabilization. The patient denied previous history of stroke, bone, or urologic pathology. He also stated that he had not suffered any head or neck injury prior the accident.

The patient was first observed in our clinic 2 years after the accident. He was in a wheelchair. Neurological examination showed marked postural instability, no rest and postural tremor, finger tapping slowed on the right, spastic tetraparesis (ASIA D) – predominantly on the left side, brisk deep tendon reflexes in the upper and lower extremities, and bilateral extensor plantar response. He also presented with vertical gaze restriction, mild hypometria in horizontal saccades, moderate dysphagia, and dysphonia. In addition, he complained of urinary urgency and polyuria, but no fecal incontinence.

**Figure 1:**
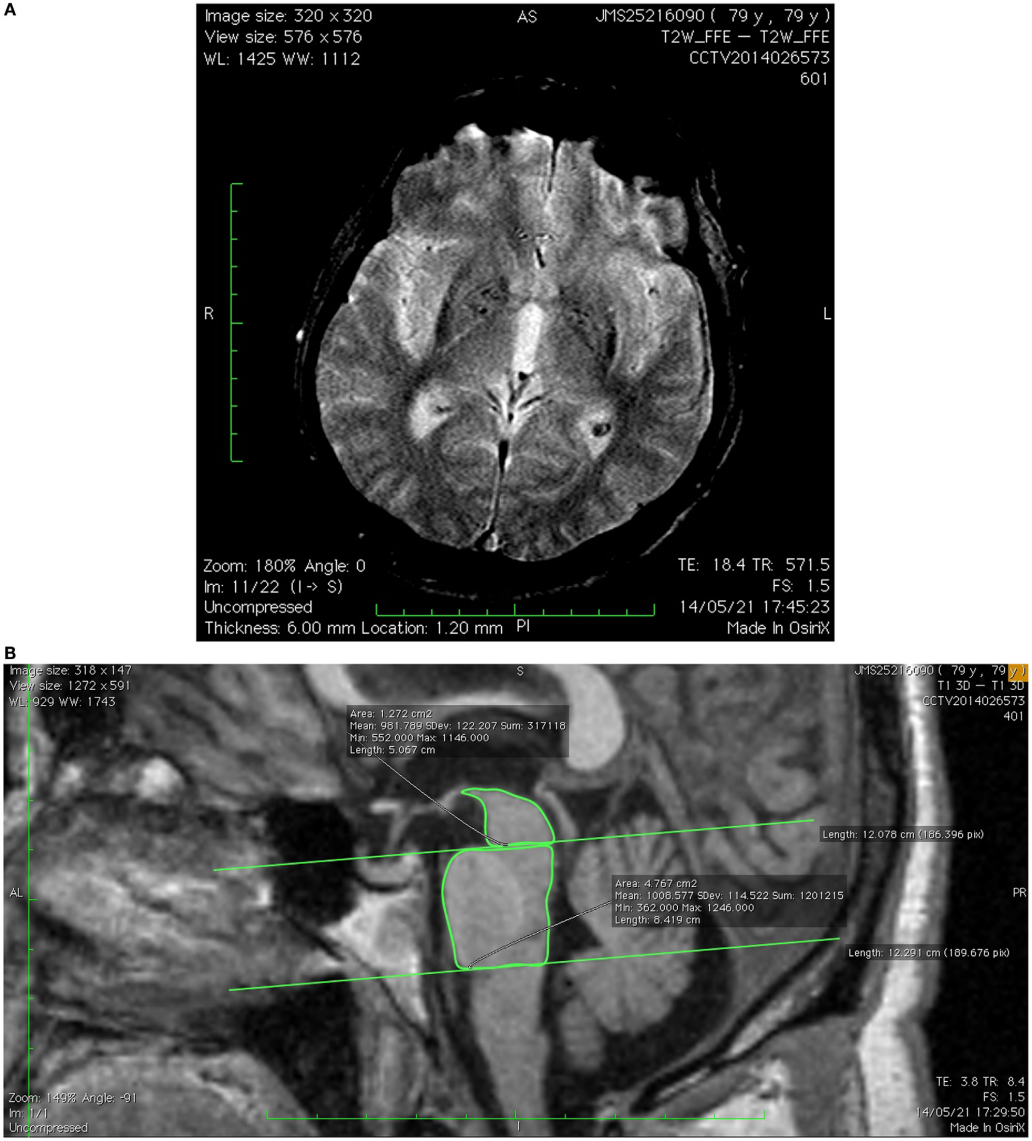
**(A)** Axial T2* image showing slit-hyperintensity in the posterolateral border of the putamen associated with increased signal and accentuation of iron deposition in putamen. **(B)** Sagittal T1-3D image showing pontine atrophy and reduction of pons/midbrain area ratio and parkinsonism index.

As atypical parkinsonism was suspected, a brain MR examination was solicited (Figure 1) and showed a combination of marked hypointensity on T2/T2* in the lateral putamen (corresponding to iron deposition) associated with a linear T2 and FLAIR hyperintensity in the posterolateral border of the putamen. There was an additional reduction of the dimensions of the pons and middle cerebellar peduncles, resulting in a decrease of the parkinsonian index. The conjunction of these findings was suggestive of parkinsonian-type MSA (MSA-P). The patient was treated with levodopa/carbidopa (600 mg/day), amantadine (100 mg/day), and baclofen (30 mg/day) and was injected with botulinum toxin in left upper limb. He was referred for neurorehabilitation and speech therapy.

## Background

Graham and Oppenheimer first coined the term multiple system atrophy (MSA) in 1969 (1). It was defined as a sporadic, neurodegenerative disorder of undetermined etiology and characterized clinically by parkinsonism, cerebellar, autonomic, and corticospinal features of variable severity (2, 3). It is one of the atypical parkinsonian syndromes and is a rare disease with prevalence rates ranging from 1.9 to 4.4/100,000 (4). When the presentation of MSA is only parkinsonism, the disease might be difficult to differentiate from PD in the first 6 years (3). The “red flag” features suggestive of MSA-P include early postural instability, rapid progression, abnormal postures, bulbar and respiratory dysfunction, emotional incontinence, early autonomic dysfunction, and poor response to levodopa (5). Köllensperger and colleagues (5), in their cohort, found that early postural instability, during the first 3 years, was present in 68% of patients, corresponding to the most frequent feature of MSA-P.

## Discussion

In our patient described above, parkinsonism features were contaminated by additional spinal cord lesion, which made the diagnosis difficult to establish. The presence of signs above the cervical lesion, such as early loss of postural reflexes, dysphagia, and dysphonia plus the poor response to levodopa, were clinical clues that led us to suspect atypical parkinsonism. Several studies (6) have proposed that MR signal changes and atrophy in putamen and infratentorial structures are suggestive of MSA-P. In our case, the presence of these features contributed to the diagnosis.

Postural dysfunction is one of the most incapacitating clinical features of MSA. It occurs early and progresses more rapidly than in PD (7, 8). Tison and colleagues (7) compared parkinsonian features in matched-samples of MSA and PD patients at initial observation and after a mean of 62 months follow-up. They observed that postural instability was more frequent in MSA at the beginning and during disease progression. Postural instability is due to the inability of the postural muscles to respond rapidly and with enough magnitude to destabilizing forces. This instability compromises the ability to maintain balance during everyday tasks, resulting in frequent falls and loss of independence (9). In our case, we speculate that the loss of postural reflexes did not allow the patient to have an effective reaction response to a low-energy trauma, resulting in a more severe injury.

## Concluding Remarks

To the best of our knowledge, no previous association in literature was made to spinal cord trauma, as an early manifestation of postural reflexes impairment, to neurodegenerative disorders.

Postural dysfunction, due to loss of reflexes, induces early clinical impairment in patients with MSA. In our case, we speculate that the loss of postural reflexes did not allow the patient to have an effective reaction response to a low-energy trauma, resulting in a more severe injury.

Severe spinal lesions caused by minor accidents can be an early sign of postural instability, which may lead to clinical suspicion of neurodegenerative disorders, including MSA.

## Consent

Written informed consent was obtained from the patient for publication of this case report and any accompanying images. A copy of the written consent is available for review by Editor-in-Chief of this Journal.

## Author Contributions

MB contributed to the conception and handling of data, clinical assessment, and writing of the first draft of manuscript; SR made clinical assessment and was involved in revising the manuscript; DS was involved in drafting the manuscript. RC agreed to be accountable for all aspects of the work in ensuring that questions related to the accuracy or integrity of any part of the case report are appropriately resolved and has given final approval of the version to be published. JF contributed to the conception, handling of data, clinical assessment, critically revising the manuscript for important intellectual content and gave final approval of the version to be published.

## Conflict of Interest Statement

The authors declare that the research was conducted in the absence of any commercial or financial relationships that could be construed as a potential conflict of interest.

## Abbreviations

MSA, multiple system atrophy; MSA-P, parkinsonian-type MSA; PD, Parkinson’s disease.
